# Video Game Training Enhances Visuospatial Working Memory and Episodic Memory in Older Adults

**DOI:** 10.3389/fnhum.2016.00206

**Published:** 2016-05-06

**Authors:** Pilar Toril, José M. Reales, Julia Mayas, Soledad Ballesteros

**Affiliations:** ^1^Studies of Aging and Neurodegenerative Diseases Research Group, Universidad Nacional de Educación a Distancia (UNED)Madrid, Spain; ^2^Department of Basic Psychology II, Universidad Nacional de Educación a Distancia (UNED)Madrid, Spain; ^3^Department of Methodology of the Behavioral Sciences, Universidad Nacional de Educación a Distancia (UNED)Madrid, Spain

**Keywords:** brain plasticity, cognitive aging, episodic memory, training, video games, visuospatial working memory

## Abstract

In this longitudinal intervention study with experimental and control groups, we investigated the effects of video game training on the visuospatial working memory (WM) and episodic memory of healthy older adults. Participants were 19 volunteer older adults, who received 15 1-h video game training sessions with a series of video games selected from a commercial package (*Lumosity*), and a control group of 20 healthy older adults. The results showed that the performance of the trainees improved significantly in all the practiced video games. Most importantly, we found significant enhancements after training in the trained group and no change in the control group in two computerized tasks designed to assess visuospatial WM, namely the Corsi blocks task and the Jigsaw puzzle task. The episodic memory and short-term memory of the trainees also improved. Gains in some WM and episodic memory tasks were maintained during a 3-month follow-up period. These results suggest that the aging brain still retains some degree of plasticity, and that video game training might be an effective intervention tool to improve WM and other cognitive functions in older adults.

## Introduction

Age-related brain changes occurring mainly in the prefrontal cortex and the medial temporal lobe system (including the hippocampus and the cerebellum) are associated with cognitive declines (Raz et al., 2005) in several functions, including processing speed (Salthouse, 1996), peripheral vision (Muiños and Ballesteros, 2014), dynamic visual acuity (Muiños and Ballesteros, 2015), working memory (WM), executive control functioning, and episodic memory (e.g., Baltes and Lindenberger, 1997; Hoyer and Verhaeghen, 2006; Nilsson, 2003; Park and Gutchess, 2002; Rönnlund et al., 2005). However, other cognitive functions, including implicit memory, verbal abilities and world knowledge, are mostly spared with age (e.g., Park et al., 2002; Mitchell and Bruss, 2003; Craik and Bialystok, 2006; Osorio et al., 2010; Ballesteros et al., 2013; Ballesteros and Mayas, 2015). Experience-related changes induced by the modification of the social environment, physical activity, and cognitive training affect brain structure and function (for a recent review see, Ballesteros et al., 2015a). Research on brain plasticity in older adults and its relationship to experiential changes is currently attracting substantial public interest (Raz and Lindenberger, 2013). However, several studies have found brain plasticity not only in healthy older adults, but also in patients suffering chronic traumatic brain injury (Sacco et al., 2011), schizophrenia (Fisher et al., 2010), and intellectual disability (Söderqvist et al., 2012). Recently, research on training-induced changes in brain and behavior has attracted a great interest in cognitive neuroscience across lifespan, especially in the older age. Studies on this field might contribute to improve our knowledge on brain plasticity and result of a great help for designing effective interventions (see Karbach and Schubert, 2013).

Training intervention studies suggest that the older human brain maintains a certain level of neural plasticity (Bialystok and Craik, 2006; Li et al., 2006, 2008). Based on this idea, an active line of research concerns ways of maintaining and/or improving cognitive skills (Green and Bavelier, 2003), delaying cognitive and brain declines as much as possible (Hertzog et al., 2008; Park and Reuter-Lorenz, 2009; Park and Bischof, 2013; Reuter-Lorenz and Park, 2014). The observed increase in neural volume in response to cognitive training is an indicator of brain plasticity (see, Boyke et al., 2009; Park and Bischof, 2013). Based on the assumption that the older brain retains at least some degree of plasticity and still has the capacity to modify its structural and functional patterns to meet new environmental demands, researchers are intensively exploring different types of intervention for older adults.

One of the most popular computerized intervention approaches is training older adults with video games. Some intervention studies have reported improvements in the trained group but not in the control group in several cognitive functions, including processing speed (e.g., Clark et al., 1987; Dustman et al., 1992; Ballesteros et al., 2014), visuo-motor coordination (Drew and Waters, 1986), attention (e.g., Goldstein et al., 1997; Belchior, 2008; Mayas et al., 2014), memory (e.g., Craik et al., 2007; Smith et al., 2009; Hampstead et al., 2012), WM (e.g., Edwards et al., 2005; Erickson et al., 2007; Anguera et al., 2013), and global cognitive function (Torres, 2008). By contrast, other studies have failed to find any positive effects of training with video games on cognition (e.g., Ackerman et al., 2010; Owen et al., 2010; Boot et al., 2013a).

Video games are designed with two aims: enjoyment and sustained player engagement (Anguera and Gazzaley, 2015). They can be classified as simple (non-action games) and complex (action games). Complex video games are fast, intense, unpredictable, and require more perceptual and cognitive skills than non-action games (e.g., Green and Bavelier, 2003; Feng et al., 2007). Some researchers have used complex video games to train older adults (e.g., Basak et al., 2008; Stern et al., 2011), while others have used non-action games, which seem more appropriate for older adults (e.g., Torres, 2008; van Muijden et al., 2012; Ballesteros et al., 2014).

An important question in this context is whether training with video games transfers to other untrained cognitive functions. This is a critical issue for its practical significance, but remains debatable. A systematic review conducted to examine the effectiveness of computer-based interventions in cognitively healthy older adults found that video game training improved processing speed and global cognition but was less efficient for improving executive functions (Kueider et al., 2012). More recently, we conducted a meta-analysis to examine the hypothesis that training older adults with video games enhances their cognitive functioning (Toril et al., 2014). The studies included in this meta-analysis were 20 experimental video game training interventions with pre- and post-training measures, published between 1986 and 2013. The mean effect size was moderate [0.37 (SE 0.05) with a 95% CI of between 0.26 and 0.48]. The results indicated that training older adults with video games produces moderate positive effects on several cognitive functions (e.g., reaction time (RT), attention, memory and global cognition), but does not improve executive functions. This meta-analytic study (see also Lampit et al., 2014) also found that these positive results were moderated by variables such as the age of the trainees and the frequency or length of the training program (the amount of time needed to induce cognitive improvement).

WM is a capacity-limited system that stores and processes information needed for ongoing cognition. This capacity-limited workspace is necessary to keep things in mind while performing complex tasks such as comprehension and reasoning (Baddeley and Hitch, 1974). This key component of cognition, central to many cognitive functions, including concentration, problem solving, and impulse control, declines significantly with age (e.g., Park et al., 2002; Bopp and Verhaeghen, 2005; Park and Reuter-Lorenz, 2009). Many recent reviews and longitudinal computerized cognitive training studies have investigated the effectiveness of computerized training approaches aimed at improving WM (e.g., Dahlin et al., 2008; Perrig et al., 2009; Klingberg, 2010; Shipstead et al., 2010, 2012; Takeuchi et al., 2010; Boot et al., 2011; Morrison and Chein, 2011). Unfortunately, the results of these studies are at best mixed, with some articles reporting the effectiveness of WM training (e.g., Borella et al., 2010; Klingberg, 2010; Morrison and Chein, 2011), while others concluded that it is ineffective (e.g., Shipstead et al., 2010; Redick et al., 2013; Ballesteros et al., 2014).

Several recent meta-analytic studies (Karbach and Verhaeghen, 2014; Lampit et al., 2014; Toril et al., 2014) have noticed the great variability in the interventions in terms for example of the intensity and duration of the training regimes, whether they are carried out at home or in the presence of the trainer, and the age of the participants. Our meta-analytic study (Toril et al., 2014) showed that a small number of training sessions is more effective than a large number, possibly because older adults get tired and lose motivation after many training sessions. We also found that the benefits of training increased with the age of participants. The lower baseline scores of the older participants can explain this result. Lampit et al. (2014) also found that computerized cognitive training can improve the cognitive performance of healthy older adults, but that its effectiveness varies across domains. They suggested that training more than three times per week is ineffective. Karbach and Verhaeghen (2014) examined the effects of executive-function and WM training in older adults, suggesting that the inconsistencies of the results were due to differences in the type, intensity, and duration of the intervention, and to the methods used to compare different studies. Their results suggest that WM and executive-function training produces significant and large improvements in the performance of the trained tasks and reliable small to medium-sized transfer effects in the process trained, at least in healthy older adults.

A recent randomized controlled trial study conducted to investigate the effects of training older adults with non-action video games on a series of cognitive functions that decline with age and on subjective wellbeing (Ballesteros et al., 2014) found significant improvements in the experimental group after training in processing speed, attention, immediate and delayed visual recognition memory, as well as a tendency to improve in some dimensions (affection and assertiveness) of the wellbeing scale. However, visuospatial WM and executive control did not improve after training. Overall, these pre-/post-training results support the view that training older adults with non-action video games improves some cognitive abilities but not others. Moreover, the assessment conducted after a 3-month no-contact interval showed that the benefits in processing speed, attention and long-term memory vanished and that only the effects on wellbeing were maintained 3 months later (see Ballesteros et al., 2015b). However, participants in the trained group showed no transfer to either executive control or spatial WM from pre-test to 3-month follow-up. These results suggest that cognitive plasticity can be induced in healthy older adults, but that periodic boosting sessions are needed to maintain the training benefits.

In view of the importance of WM for the daily life activities of older adults, we designed this longitudinal intervention study taking into account the results of our previous study (Ballesteros et al., 2014, 2015b) and the findings of several recently published meta-analyses (Karbach and Verhaeghen, 2014; Lampit et al., 2014; Toril et al., 2014). The program was composed of 15 1-h sessions. An important variable is the number of video games included in the training schedule (see Toril et al., 2014), and we therefore selected just six non-action video games to train mainly WM. Importantly, all the participants in the experimental group were trained in group sessions at the municipal senior center and in the presence of an experimenter (as recommended by Kelly et al., 2014 and Lampit et al., 2014).

The goal of the present study was to investigate whether cognitively healthy older adults could benefit from training with non-action video games. We addressed two main questions. First, would training older adults with non-action video games improve their visuospatial WM as well as short- and long-term memory? Secondly, would any improvements persist after a 3-month no-contact period? Based on the results of previous studies, we hypothesized that: (1) video game training would improve the visuospatial WM of older adults; (2) the effects of training would transfer to episodic memory; and (3) memory improvements would persist 3 months after finishing the training program.

## Materials and Methods

### Participants

Forty cognitively healthy volunteer older adults were recruited from a municipal senior center in the Madrid suburbs to participate in this training study. They all had normal or corrected-to-normal vision and hearing and informed that they did not have previous experience with video games. After signing a consent form, participants were randomly assigned either to the trained (experimental) group or to the control group. Participants in both groups regularly attended cultural activities at the senior center (e.g., painting classes, lectures, cultural visits). The control group continued their routine lifestyle activities at the senior center. The study was approved by the Ethics Committee of the Universidad Nacional de Educación a Distancia. The inclusion criteria were to obtain a score of 26 or above on the Mini-Mental State Examination (MMSE; Folstein et al., 1975) and a normal score on the Information subscale of the Wechsler Adult Intelligence Scale (WAIS III; Wechsler, 1999). The two groups did not differ in age, years of education, or in the Information subscale and MMSE scores (all *p*s > 0.05). In the experimental group, one participant declined to participate after screening for medical reasons. Thus, 19 participants in the training group and 20 in the control group completed the study. Demographic data for each group are displayed in Table 1.

**Table 1:** **Demographic information about participants in each group**.

Characteristics	Experimental mean (SD)	Control mean (SD)	*p*	*F*
Age (Years)	69.95 (6.73)	73.20 (6.48)	0.13	2.36
Education (Years)	13.37 (3.27)	12.85 (3.36)	0.62	0.23
MMSE	28.31 (1.00)	27.75 (1.48)	0.17	1.92
Information	18.42 (2.61)	16.95 (3.06)	0.11	2.58

*Note: Means and Standard deviations (SD) by group; MMSE, Mini-Mental State Examination*.

### Study Design

The study was a 2 (group: experimental, control) × 3 (session: pre-training, post-training, 3-month follow-up) mixed factorial design. Group was the between-subjects factor and session was the within-subjects factor. To investigate the effectiveness of the intervention to improve and/or maintain both visuospatial WM and short- and long-term memory, participants performed a series of tests and experimental tasks designed to assess these types of memory: digit span forward and backward, Corsi blocks, Jigsaw puzzle task, and immediate and delayed visual episodic memory tasks (Faces I and II, and Family Pictures I and II from Wechsler Memory Scale, WMS III). The Corsi blocks and the Jigsaw puzzle tasks were programmed using E-Prime 2.0 (Psychology Software Tools Inc., Pittsburg, PA, USA). Long-term memory was assessed with tests from the WMS-III (Wechsler, 1997), and the Digit Span tasks were extracted from WAIS III (Wechsler, 1999).

### Training Schedule and Overview of the Training Program

Participants assigned to the experimental group completed 15 1-h training sessions at the community senior center in the presence of the experimenter over a period of 7–8 weeks. In each training session, participants played six video games twice each. The games were selected from *Lumosity*^1^, a web-based cognitive training platform^2^; they were *Speed Match, Memory matrix, Rotation matrix, Face memory, Money comb* and* Lost in migration*. The session score for each participant on each game was calculated as the mean score of the first and second time they played the game. The session RT for each participant on each game was calculated as the mean performance of the first and second time they played the game. The control group did not receive training but met the experimenter periodically (once a month) in the senior center to talk about their activities and other general topics related to aging. The video games used in this study are described below.

#### Speed Match

In this game, a symbol is displayed on the computer screen, followed immediately by another. The trainee has to decide whether the two symbols are the same, indicating their choice by pressing one of two keys (same, different) as fast as possible.

#### Memory Matrix

A matrix varying in size is displayed in the center of the screen with a pattern of colored squares followed by a blank matrix. The player has to reproduce the pattern by clicking on the squares that were colored.

#### Rotation Matrix

This game is similar to the previous one, except that the matrix is rotated between the coding phase and the response phase. The player has to mentally rotate the encoded matrix, and click on the correct positions of the colored squares.

#### Face Memory

Different faces appear on the screen continuously, one after another, and the player has to decide whether the face on the screen matches the one shown one (1-back), two (2-back), or three (3-back) faces before.

#### Moneycomb

In this game, a honeycomb appears in the center of screen and a sequence of tokens of different values is presented briefly inside it. The task consists of clicking on the correct tiles of the honeycomb to reveal the tokens in the correct order (from lowest to highest value).

#### Lost in Migration

In this game, a static flock of birds appears in the center of the screen. The goal is to identify the direction in which the bird in the middle of the flock is flying (right, left, upward, downward) by pressing one of the four arrow keys on the keyboard as fast as possible.

Participants received points based on their performance on each video game. Some of the games also recorded response times. None of the participants in the study reported that they had any previous experience of playing video games.

### Assessment Tasks and Procedures

Assessment tasks fell into one of the following three domains: visuospatial WM, short-term memory and episodic memory.

### Visuospatial Working Memory Tasks

Visuospatial WM (Baddeley and Hitch, 1974) was assessed with the Corsi blocks and the Jigsaw-puzzle task.

#### Corsi Blocks Task

The original Corsi Blocks task (Milner, 1971) consisted of a set of nine identical blocks (3 cm × 3 cm × 3 cm) unevenly positioned on a wooden board (23 cm × 28 cm). The participant had to point to the blocks in their order of presentation. The length of the sequence increased until recall was no longer correct in terms of order or position (Berch et al., 1998). In this study, we used the same computerized version of the Corsi task as in our previous study (Ballesteros et al., 2014) with four levels of increasing difficulty (2, 3, 4 and 5 cube positions) and 10 trials per level. The stimuli were black squares on a 3 × 3 matrix that appeared one after the other, for 1 s each. The positions in each sequence were selected randomly, with the restriction that stimuli could not appear in the same position in two consecutive sequences. In each trial, the participant reproduced the previously presented sequence of cubes (the black squares in the 3 × 3 matrix) by writing down their order of presentation on a separate response sheet. To familiarize participants with the task, they performed a practice block of trials. The final score was the proportion of correct sequences obtained at each difficulty level.

#### Jigsaw-Puzzle Task

The original pencil-and-paper Jigsaw-Puzzle task was developed to assess active visuospatial abilities (Richardson and Vecchi, 2002). We designed a computerized version of this task with puzzles consisting of 4, 6 or 9 pieces. Each piece was numbered and the participant had to write down on a response sheet the number corresponding to the pieces in the correct spatial positions. The stimuli were 15 pictures with similar visual complexity (mean = 2.4, SD = 0.32) and familiarity (mean = 4.3, SD = 0.26) selected from the Snodgrass and Vanderwart (1980) picture set. Each picture was fragmented into 4, 6 and 9 pieces to produce 45 different puzzles. The pictures were enlarged to fit a 12 cm × 12 cm area and were cut into four 6 cm × 6 cm pieces, six 6 cm × 4 cm pieces, or nine 3 cm × 3 cm pieces using Adobe Photoshop CC (Adobe Systems Software, Ireland Ltd.). We generated three different counterbalanced orders. Different pictures were used at pre-test, post-test and follow-up assessments. Participants were presented with 15 puzzles representing all possible combinations and number of pieces. The response sheets contained grids of the same size as the original pictures with the appropriate number of squares (4, 6, 9 squares). We used two puzzles as practice items and their results were not included in the analysis. For each trial, a fragmented picture appeared on the computer screen and the participant wrote down on the response sheet the appropriate numbers to form a spatially correct picture. The jigsaw was presented on the computer screen for 90 s. Participants were allowed to correct errors within that time. Performance was assessed in terms of the proportion of correct puzzles per level (4, 6 and 9 pieces).

### Short-Term Memory

Short-term memory was assessed with the Digit Span Test of the WAIS III scale (Wechsler, 1999).

#### Digit Span Test

This test has two parts: Digit span forward and Digit span backward*.* For each part, the test administrator says a series of numbers aloud at the rate of one per second. The participant then repeats the numbers in the same order (digit span forward) or in reverse order (digit span backward). Both tests begin with a series of two numbers. For digits forward, the test continues up to a maximum of eight numbers. For digits backward, the test continues up to a maximum of seven numbers. Participants are given two trials at each length and the test continues until the participant fails both trials at one length. In both the forward and the backward task, the score was the maximum number of correctly remembered digits.

#### Immediate and Delayed Episodic Memory Tests

The **Faces and Family Pictures** subtests of the WMS-III were used to assess visual episodic memory. For immediate recognition and inmediate recall, we used Faces I and Family Pictures I, respectively. Delayed recognition and recall was assessed 25 min later using Faces II for delayed recognition and Family Pictures II for delayed recall.

## Results

### Video Game Practice Effects

**Figure 1:**
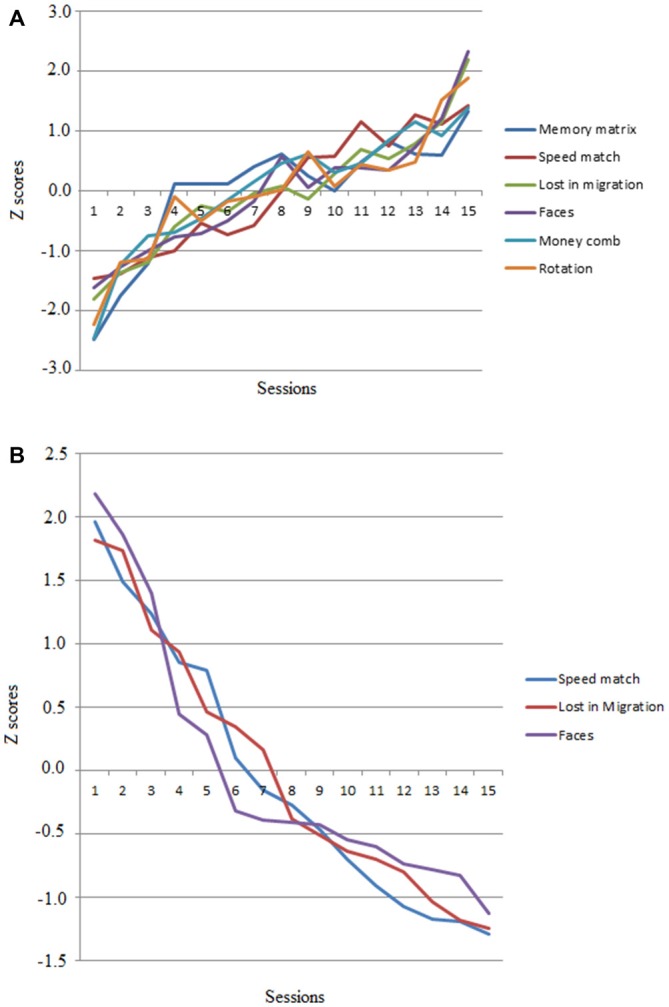
**(A)** Average performance scores obtained in the six non-action video games across the 15 sessions in *Z* scores (mean 0; standard deviation 1). **(B)** Average response times of four video games across the training sessions in *Z* scores.

Although the main dependent variables of this intervention study were the scores obtained on the memory tests, we also analyzed performance on the video games to evaluate whether the trained participants improved as a consequence of playing the video games. Video game performance showed significant improvements (accuracy and response times) across the 15 training sessions (see Figures 1A,B). Figure 1A shows the positive linear trend of the mean number of correct responses as a function of session. Figure 1B presents the mean response times for the video games that recorded response times. The mean scores of each game at the beginning and end of the training period were compared using regression analysis, with Training Session as the predictor variable and RT and Game Score as the criterion variables. Performance on all games improved after training. *R*^2^ coefficients were high and accounted for more than 80% of the variance of the model in the six games. The ANOVAS for the previous analyses showed that all *R*^2^ coefficients were statistically significant. This means that Training Session was a reliable predictor of Score and RT in the six games. Table 2 summarizes the results.

**Table 2:** **Determination coefficients (*R*^2^), *F* and *p* values for the six video games**.

Videogame	*DV*	*R*^2^	*R*^2^ (corr)	*F*	*p*
Memory matrix	Score	0.699	0.675	30.14	0.00
Speed match	Score	0.952	0.949	260.64	0.00
	RT	0.951	0.948	254.91	0.00
Lost in migration	Score	0.921	0.914	150.65	0.00
	RT	0.954	0.951	271.49	0.00
Faces	Score	0.896	0.888	112.42	0.00
	RT	0.813	0.798	56.44	0.00
Money comb	Score	0.866	0.856	84.34	0.00
Rotation matrix	Score	0.840	0.828	68.45	0.00

*Note. DV, Dependent variable; R^2^, Regression coefficient; R^2^corr, corrected regression coefficient; F, F values of ANOVAs; p, p values*.

### Effects of Video Game Training on Visuospatial Working Memory Tasks and Other Memory Tests

We investigated whether training with video games improved memory abilities that decline with age, especially visuospatial WM and episodic memory. We addressed two questions. The first was whether training older adults with video games would transfer to performance on a series of memory tasks (transfer effects). The second was whether these possible enhancements would remain after a 3-month no-contact period (maintenance). To answer these questions, we investigated whether group (trained vs. control) interacted with session (pre-test, post-test, follow-up) with regard to performance on the different memory tests. Statistical analyses were conducted using Bonferroni correction for main effects and interactions in all tasks.

### Jigsaw-Puzzle Task

**Figure 2:**
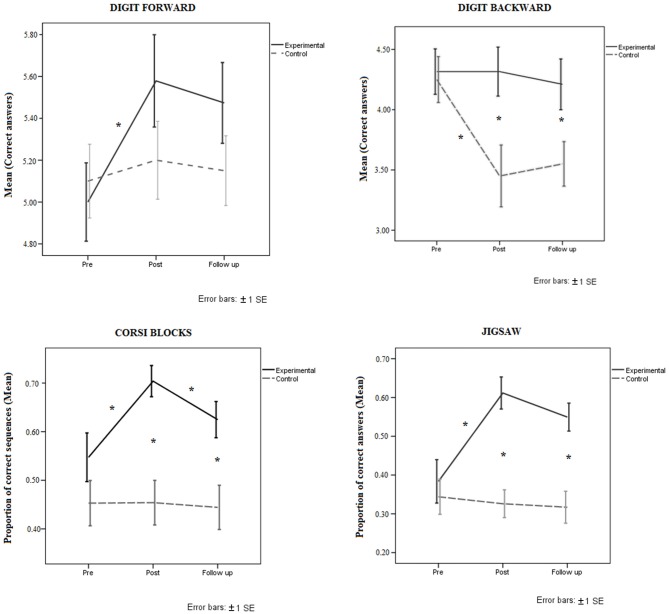
**Top:** Mean performance of trained and control groups at pre-test, post-test and follow-up assessments in Digits forward (left) and Digits backward (right). **Bottom:** Mean performance of trained and control groups at pre-test, post-test and follow-up assessments in the working memory (WM) tasks (left: Corsi blocks; right: Jigsaw puzzle tasks). Error bars represent ± SE. *p < 0.05.

A Group (2) × Session (3) × Level of fragmentation (3) mixed ANOVA with Group as the between-subjects factor and Session and Level of fragmentation as the within-subjects factor was performed on the proportion of correct puzzles completed at each level of fragmentation. The results showed that the main effect of Group was statistically significant (*F*_(1,37)_ = 12.10, *MSe* = 3.03, *p* = 0.001, $\eta_{p}^{2}$ = 0.84). The trained group performed better (mean = 0.52, SD = 0.17) than the control group (mean = 0.33, SD = 0.17). Session was also statistically significant (*F*_(2,74)_ = 8.71, *MSe* = 0.32, *p* = 0.001, $\eta_{p}^{2}$ = 0.19). Participants performed better at post-test (mean = 0.41, SD = 0.12) than at pre-test (mean = 0.36, SD = 0.18), but there was no difference between post-test and 3-month follow-up (*p* = 0.29). Level of fragmentation was also significant (*F*_(2,74)_ = 207.56, *MSe* = 12.70, *p* = 0.001, $\eta_{p}^{2}$ = 0.84), showing that performance deteriorated with higher levels of fragmentation (level 4, mean = 0.75, SD = 0.18; level 6, mean = 0.43, SD = 0.24; level 9, mean = 0.09, SD = 0.12). The two-way Session by Group interaction was statistically significant (*F*_(2,74)_ = 13.30, *MSe* = 0.49, *p* = 0.001, $\eta_{p}^{2}$ = 0.26), showing that the trained group performed better at post-test (mean = 0.61, SD = 0.13) than the control group (mean = 0.32, SD = 0.13). The trained group performed better (mean = 0.55, SD = 0.13) at the 3-month follow-up assessment (*p* = 0.001) than the control group (mean = 0.32, SD = 0.13), while the performance of the control group did not differ between sessions. No other interaction was significant (all *p* > 0.05); see Figure 2 (bottom right).

### Corsi Blocks Task

A Group (2) × Session (3) × Corsi level (2, 3, 4 and 5 blocks) mixed ANOVA with Group as the between-subjects factor and Session and Corsi level as within-subjects factors were conducted on the proportion of correct sequences per level. The results showed that the main effect of Group was statistically significant (*F*_(1,37)_ = 10.04, *MSe* = 3.55, *p* = 0.001, $\eta_{p}^{2}$ = 0.21), showing that the trained group (mean = 0.62, SD = 0.17) outperformed the control group (mean = 0.45, SD = 0.17). The main effect of Session was also statistically significant (*F*_(2,74)_ = 5.43, *MSe* = 0.24, *p* = 0.001, $\eta_{p}^{2}$ = 0.12), with better performance at post-test (mean = 0.58, SD = 0.12) than at pre-test (mean = 0.50, SD = 0.18). There were significant differences between pre-test and post-test assessments (*p* = 0.01). Moreover, there were marginally significant differences (*p* = 0.054) between post-test (mean = 0.58, SD = 0.12) and 3-month follow-up (mean = 0.53, SD = 0.12), while performance at pre-test and 3-month follow-up (*p* = 0.57) did not differ. The main factor of Corsi level was also significant (*F*_(3,37)_ = 285.84, *MSe* = 12.84, *p* = 0.001, $\eta_{p}^{2}$ = 0.88), showing that performance deteriorated as the number of blocks increased (Corsi 2, mean = 0.89, SD = 0.06; Corsi 3, mean = 0.67, SD = 0.18; Corsi 4, mean = 0.46, SD = 0.24; Corsi 5, mean = 0.12, SD = 0.12). The two-way Session by Group interaction was also statistically significant (*F*_(2,74)_ = 5.25, *MSe* = 0.23, *p* = 0.001, $\eta_{p}^{2}$ = 0.12). The analysis of this interaction showed that there were significant differences (*p* = 0.001) between pre- and post-test in the trained group (mean pre-test = 0.55, SD = 0.21; mean post-test = 0.70, SD = 0.17), but a reverse trend was observed in the control group (*p* = 1.00). There were also significant differences (*p* = 0.01) in the trained group between post-test and 3-month follow-up (mean post-test = 0.70, SD = 0.17; mean follow-up = 0.62, SD = 0.17), with lower performance at the 3-month follow-up. Differences between pre- and post-test were not significant (*p* = 1.00) in the control group (mean pre-test = 0.45, SD = 0.22; mean post-test = 0.46, SD = 0.17). The two-way Group by Level interaction was also significant (*F*_(3,111)_ = 5.67, *MSe* = 0.24, *p* = 0.001, $\eta_{p}^{2}$ = 0.13). The analysis of this interaction showed that there were significant differences (*p*s < 0.05) between groups at all Corsi levels. In the trained group, the means for each level were: level 2 = 0.94, SD = 0.08; level 3 = 0.78, SD = 0.17; level 4 = 0.60, SD = 0.26; level 5 = 0.17, SD = 0.13. In the control group the means were: level 2 = 0.84, SD = 0.08; level 3 = 0.54, SD = 0.17; level 4 = 0.32, SD = 0.26; level 5 = 0.07, SD = 0.13). Although the trained group performed better than the control group in this task, performance deteriorated in both groups as the number of blocks increased; see Figure 2 (bottom left).

### Digit Forward Test

The ANOVA conducted with Group (2) and Session (3) on the numbers of correct digits reportedly showed that Session was significant (*F*_(2,74)_ = 3.97, *MSe* = 1.23, *p* = 0.02, $\eta_{p}^{2}$ = 0.09), with better performance at post-test (mean = 5.38, SD = 0.87) than at pre-test (mean = 5.05, SD = 0.74), but there were no significant differences between post-test and 3-month follow-up (*p* = 1.00). The trained group performed better at post-test than at pre-test (*p* = 0.02), but there was no difference between post-test and 3-month follow-up (*p* = 1.00). Group as a main factor was not significant (*p* = 0.37), as the performance of the trained group (mean = 5.35, SD = 0.69) was similar to that of the control group (mean = 5.15, SD = 0.71). No other factors or interactions were significant (all *p*s > 0.05); see Figure 2 (top left).

### Digit Backward Test

An ANOVA with Group (2) and Session (3) was conducted on the numbers of correct digits reported. The main factor of Group was significant (*F*_(1,37)_ = 5.09, *MSe* = 8.78, *p* = 0.03, $\eta_{p}^{2}$ = 0.12), showing that the trained group performed better (mean = 4.29, SD = 0.73) than the control group (mean = 3.75, SD = 0.67). The main effect of Session (*F*_(2,74)_ = 5.24, *MSe* = 1.96, *p* = 0.001, $\eta_{p}^{2}$ = 0.12) was significant. Performance at pre-test was better than at post-test (mean pre-test = 4.28, SD = 0.81; mean post-test = 3.88, SD = 0.93). The control group performed worse at post-test than at pre-test. The two-way Group by Session interaction was also significant (*F*_(2,74)_ = 4.69, *MSe* = 1.75, *p* = 0.01, $\eta_{p}^{2}$ = 0.11). Simple effects analysis showed no significant differences in the trained group between pre-test, post-test and 3-month follow-up evaluations (*p* = 1.00). Participants performed similarly in the three assessment sessions (mean = 4.31, SD = 0.82; mean = 4.31, SD = 0.80; mean = 4.26, SD = 0.87, for pre-test, post-test and 3-month follow-up, respectively). By contrast, there were significant differences in the control group between pre- and post-test (*p* = 0.001) due to poorer performance at post-test (mean post-test = 3.45; SD = 1.14) than at pre-test (mean = 4.25, SD = 1.14), but there were no differences (*p* = 1.00) between post-test and 3-month follow-up; see Figure 2 (top right).

### Episodic Memory Test (Faces)

**Figure 3:**
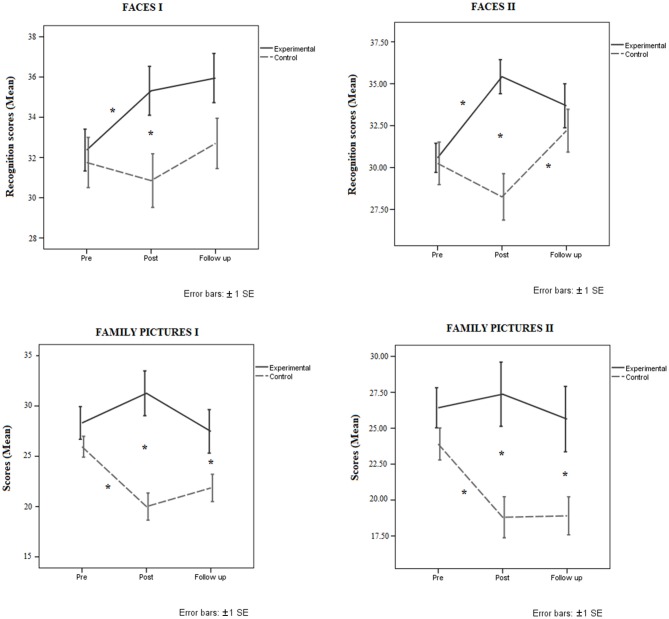
**Mean performance of trained and control groups at pre-test, post-test and follow-up in the episodic memory tasks. Top:** Faces I (left) and Faces II (right). **Bottom:** Family Pictures I (left) and Family Pictures II (right). Error bars represent ± SE. *p < 0.05.

The results of the episodic memory tests are shown in Figure 3 and Table 3.

**Table 3:** **Pre-test, post-test and follow-up training performance on working memory and episodic memory tasks corresponding to the trained and the control groups. M (Mean), SD (Standard deviation)**.

Task	Experimental			Control		
	*Pre M (SD)*	*Post M (SD)*	*Follow up M (SD)*	*Pre M (SD)*	*Post M (SD)*	*Follow up M (SD)*
**Corsi Blocks task**
2 Serial position (Proportion)	0.86 (0.20)	0.98 (0.03)	0.95 (0.07)	0.79 (0.17)	0.86 (0.12)	0.86 (0.13)
3 Serial position (Proportion)	0.68 (0.30)	0.86 (0.13)	0.81 (0.16)	0.59 (0.24)	0.53 (0.27)	0.55 (0.29)
4 Serial position (Proportion)	0.50 (0.33)	0.73 (0.26)	0.57 (0.30)	0.35 (0.35)	0.36 (0.35)	0.28 (0.32)
5 Serial position (Proportion)	0.14 (0.21)	0.23 (0.24)	0.15 (0.21)	0.08 (0.17)	0.07 (0.18)	0.08 (0.16)
**Jigsaw puzzle task**
4 pieces (Proportion)	0.68 (0.32)	0.93 (0.11)	0.92 (0.15)	0.67 (0.28)	0.59 (0.27)	0.65 (0.23)
6 pieces (Proportion)	0.42 (0.36)	0.64 (0.21)	0.58 (0.27)	0.32 (0.34)	0.22 (0.27)	0.29 (0.35)
9 pieces (Proportion)	0.05 (0.18)	0.18 (0.21)	0.14 (0.24)	0.05 (0.14)	0.02 (0.08)	0.02 (0.06)
**Digit forward**
Score	5.00 (0.81)	5.57 (0.96)	5.47 (0.84)	5.10 (0.78)	5.20 (0.83)	5.15 (0.74)
**Digit backward**
Score	4.31 (0.82)	4.32 (0.74)	4.26 (0.82)	4.25 (0.85)	3.45 (1.14)	3.55 (0.82)
**Faces I**
Score	32.37 (4.53)	35.31 (5.30)	35.94 (5.32)	31.75 (5.58)	30.85 (5.95)	32.70 (5.59)
**Faces II**
Score	30.58 (3.79)	35.42 (4.43)	33.68 (5.71)	30.25 (5.67)	28.25 (6.19)	32.20 (5.71)
**Family Pictures I**
Score	28.16 (7.15)	31.42 (9.13)	27.47 (9.43)	25.95 (4.63)	20.00 (6.03)	21.85 (6.07)
**Family Pictures II**
Score	26.42 (6.08)	27.36 (9.74)	25.10 (9.42)	23.90 (4.98)	18.75 (6.36)	18.90 (5.91)

#### Faces I

An ANOVA with Group (2) and Session (3) was performed on the recognition scores (Faces I). The analysis showed that Group was not significant although there was a trend in that direction (*p* = 0.07). Session was significant (*F*_(2,74)_ = 4.37, *MSe* = 50.11, *p* = 0.01, $\eta_{p}^{2}$ = 0.10) with better performance at the 3-month follow-up (mean = 34.32, SD = 5.42) than at pre-test (mean = 32.05, SD = 5.05). There were no significant differences (*p* = 0.55) between pre-test (mean = 32.05, SD = 5.05) and post-test (mean = 33.08, SD = 5.61), of between post-test (mean = 33.08, SD = 5.61) and 3-month follow-up (mean = 34.32, SD = 5.42). The two-way Group by Session interaction was significant (*F*_(2,74)_ = 3.28, *MSe* = 37.67, *p* = 0.04, $\eta_{p}^{2}$ = 0.08). The analysis of this interaction showed that there were significant differences between groups at post-test (*p* = 0.01), but only a trend was found at 3-month follow-up (*p* = 0.07). The trained group performed better at post-test than at pre-test (*p* = 0.03), but with similar performance at post-test and 3-month follow-up evaluations (*p* = 1.00). By contrast, the control group performed similarly in the three evaluation sessions (*p* > 0.05).

####  Faces II

The ANOVA conducted with Group (2) and Session (3) on the recognition scores (Faces II) showed that the effect of Group was statistically significant (*F*_(1,37)_ = 4.20, *MSe* = 262.15, *p* = 0.04, $\eta_{p}^{2}$ = 0.10). The trained group performed better (mean = 33.22, SD = 4.52) than the control group (mean = 30.23, SD = 4.55). Session was also significant (*F*_(2,74)_ = 5.40, *MSe* = 62.57, *p* = 0.001, $\eta_{p}^{2}$ = 0.12). There were no differences between pre-test and post-test scores (*p* = 0.15), or between post-test and 3-month follow-up scores (*p* = 0.55), but performance was better (*p* = 0.001) at 3-month follow-up (mean = 32.94, SD = 5.61) than at pre-test (mean = 30.41, SD = 4.80). The two-way Group by Session interaction was also statistically significant (*F*_(2,74)_ = 11.29, *MSe* = 130.70, *p* = 0.001, $\eta_{p}^{2}$ = 0.23), suggesting that groups differed at post-test (*p* = 0.001), but not at the 3-month follow-up assessment (*p* = 0.42). The trained group improved from pre-test to post-test (*p* = 0.001), but not between post-test and 3-month follow-up (*p* = 0.43). Moreover, there were significant differences (*p* = 0.02) between pre-test and 3-month follow-up, suggesting that performance at follow-up (mean = 33.68, SD = 5.69) was better than at pre-test (mean = 30.57, SD = 4.82). In the control group there were no significant differences between pre-test and post-test (*p* = 0.14), but participants in this group performed better at 3-month follow-up than at post-test (*p* = 0.001). Differences between pre-test and 3-month follow-up were not significant (*p* = 0.25).

### Episodic Memory Tests (Family Pictures)

#### Family Pictures I

An ANOVA with Group (2) and Session (3) performed on the recall scores (Family Pictures I) showed a significant effect of Group (*F*_(1,37)_ = 11.18, *MSe* = 1203.86, *p* = 0.001, $\eta_{p}^{2}$ = 0.23), indicating that the trained group performed better (mean = 29.01, SD = 5.95) than the control group (mean = 22.60, SD = 5.94). Session was not significant (*p* = 0.06), but the Session × Group interaction was significant (*F*_(2,74)_ = 8.47, *MSe* = 211.37, *p* = 0.001, $\eta_{p}^{2}$ = 0.18). The analysis of this interaction suggests that there were significant differences between groups at post-test (*p* = 0.001) and 3-month follow-up (*p* = 0.03). Performance of the trained group did not differ between sessions (*p* > 0.05), but the control group performed worse at post-test than at pre-test (*p* = 0.001) with no difference between post-test and 3-month follow-up (*p* = 0.88). However, the control group performed worse at 3-month follow-up than at pre-test (*p* = 0.01).

#### Family Pictures II

The ANOVA Group (2) × Session (3) conducted on the recall scores (Family Pictures II) showed that the effect of group was significant (*F*_(1,37)_ = 8.89, *MSe* = 977.08, *p* = 0.001, $\eta_{p}^{2}$ = 0.19), suggesting that the trained group performed better (mean = 26.29, SD = 6.00) than the control group (mean = 20.51, SD = 6.03). The effect of Session was also statistically significant (*F*_(2,74)_ = 4.09, *MSe* = 100.71, *p* = 0.02, $\eta_{p}^{2}$ = 0.10). There were no significant differences between post-test and 3-month follow-up (*p* = 1.00) or between pre-test and post-test (*p* = 0.27), but scores differed significantly between pre-test and 3-month follow-up (*p* = 0.01). Performance was worse at 3-month follow-up (mean = 22.00, SD = 7.08) than at pre-test (mean = 25.16, SD = 4.90). The Group by Session interaction was significant (*F*_(2,74)_ = 3.73, *MSe* = 91.87, *p* = 0.02, $\eta_{p}^{2}$ = 0.09), showing that the trained group performed better than the control group at both post-test and 3-month follow-up assessments. The trained group did not differ between sessions (*p* > 0.05), but the control group performed worse at post-test than at pre-test (*p* = 0.01). Moreover, the control group performed similarly at post-test and 3-month follow-up (*p* = 1.00), but this group performed worse (*p* = 0.001) at the 3-month follow-up test (mean = 18.90, SD = 7.82) than at pre-test (mean = 23.90, SD = 5.54).

## Discussion

The study yielded three main findings. First, the trainees improved their video game performance across sessions. Second, and most important, the trainees performed the Jigsaw puzzle, Corsi Blocks, Digit forward, and Faces I and II tasks better than the control group. Third, the improved performance of the trained group was maintained from baseline to the 3-month follow-up for the Jigsaw puzzle task, which is a visuospatial WM task, and on the Digits forward, and Faces I and II tasks, but not on the Corsi Blocks, the other visuospatial WM task. These results are encouraging considering the age-related declines that occur in these memory functions.

### Non-Action Video Games Training Transferred to Working Memory

The improvements found in the present study are in line with previous findings reported in other video game training studies conducted with older adults (e.g., Basak et al., 2008; Anguera et al., 2013). Basak et al. (2008) found improvements on working memory tasks after training older adults for 23.5 h with a real-time strategy video game (action video game). Anguera et al. (2013) trained older adults (60–75 years) for 4 weeks with an adaptive version of *Neuroracer*. Participants reduced multitasking costs at the post-training evaluation compared to an active control group and a no-contact control group. Moreover, the benefits of training were extended to an untrained WM task, and gains persisted for 6 months.

The results of this longitudinal study are in agreement with those of other researchers who trained older adults using computerized training programs. For example, Buschkuehl et al. (2008) conducted an adaptive visual WM training study with oldest-old adults (mean age = 80 years). They found substantial gains in the trained task and improvements immediately after training in visual WM, which disappeared at the 1-year follow-up. Li et al. (2008) also investigated the effects of WM training on performance improvement, transfer and short-term maintenance of practice gains. In their study, young and older adults practiced a spatial WM task for 45 days, about 15 min per day. In both age groups, these researchers found improvements on the practiced tasks, and near transfer to spatial and numerical *n*-back tasks. Moreover, practice gains and near transfer effects were maintained at 3-month follow-up, but performance after training was lower in older than in young adults. Dahlin et al. (2008) conducted a computer–based training study with young and older adults based on updating information in WM. The results showed that both trained groups showed significantly greater improvement on the letter memory criterion task than the control group. Interestingly, gains were maintained 18 months later in young adults but not in older adults. Recently, Zinke et al. (2014) trained WM in older adults in nine sessions over 3 weeks and found near transfer effects in a Corsi blocks task at post-test compared with pre-test.

The results of the present study also agree with findings of a recent meta-analysis (Karbach and Verhaeghen, 2014). The authors showed that executive functions and WM training led to significant improvements in performance in the trained tasks as well as large near transfer effects in older adults. However, the findings of the present study do not agree with the results of Maillot et al. (2012) who found that after a 24-h training program the trainees improved more than controls in measures of executive control and processing speed functions, but not in visuospatial measures. The present results conflict with those of Ballesteros et al. (2014), who did not find any improvement in visuospatial WM tasks (Corsi Blocks and Jigsaw puzzle tasks) or executive functions after 20 1-h training sessions with 10 non-action video games selected from the *Lumosity* platform, although the video game training intervention was effective for improving RT, attention, and episodic memory.

The trained group improved digit span forward performance after training while the control group performed similarly across the three assessment sessions. However, in digit span backward test (a more difficult task), the trained group did not improve after training while the performance of the control group declined. It is important to note that the age-related declines in digit span backward performance is greater than that in digit span forward. This result is in agreement with the meta-analysis of Babcock and Salthouse (1990). This result supports the idea that with advancing age, the digit span forward test tends to remain stable while the digit span backward task tends to decline (Lezak, 1995). This might explain the performance of our control group in the forward and backward span digit tests.

### Video Game Training Enhanced Some Episodic Memory Tests

The present study also found improvements in some episodic memory tests after training, similar to the results of our previous study (Ballesteros et al., 2014), in which we found effects of training in episodic memory (Family Pictures I and II). However, in the present study we found improvements after training in Faces (I and II), which were maintained over a 3-month period without contact. We found improvements in Faces I and II (two recognition tests) after training but not in Family Pictures (two recall tests). This result might be explained because free recall requires greater resources than recognition and this effect increases with age (Craik and McDowd, 1987). In Faces, the recognition test, the information is present while performing the memory task. However, in Family Pictures, a recall test, very few cues are provided and participants have to initiate a series of mental operations, which require more effort. In the trained group, video game training could help participants to recall features although they did not improve after training. The results obtained in Faces suggest that the task was easy for both groups. The trained group improved after training and controls were able to maintain their performance over time.

Our results are in line with those of Buschkuehl et al. (2008) who conducted a WM training study with 80-year-old adults who trained twice a week for 3 months. Participants showed improvements in the trained tasks (visual WM tasks) and to a lesser degree, in a visual episodic memory task (visual free recall) in which they had to look for differences between two almost identical pictures.

### Results at 3-Month Follow-Up

The usefulness of the intervention depends on both the occurrence of transfer effects and the durability of the training effects. Accordingly, it was important to ascertain whether the benefits found at post-training on some WM tasks and short- and long-term memory tests were maintained after a 3-month no-contact follow-up period. In this study, transfer effects were maintained in the Digit forward test, the Jigsaw puzzle task, and in the Faces I and Faces II tests. However, significant improvements in the Corsi blocks task were not maintained.

Our results are in line with those of Anguera et al. (2013) who trained older adults for 4 weeks with an adaptive version of *Neuroracer* and found benefits after training. Specifically, they reported reduced multitasking costs in the trained group compared to the control group. The benefits found after training extended to an untrained WM task, and gains persisted for 6 months. The 3-month maintenance found in the present study is in line with the results of Li et al. (2008) who found specific improvements in young and older adults in a WM task and maintenance of near transfer effects at 3-month follow-up.

### The Video Games Training Debate

The conflicting results obtained in cognitive and brain-training studies with computerized cognitive exercises and video games have been explored in several recent meta-analyses (Karbach and Verhaeghen, 2014; Lampit et al., 2014; Toril et al., 2014). Specifically, we found that short training interventions conducted with older adults produced better results than long regimes (Toril et al., 2014). Training sessions are exciting at first, but older adults get tired and bored during the last sessions. Karbach and Kray (2009) found significant transfer effects in older adults after just four training sessions, as did Kramer et al. (1995) who provided a limited number of training sessions. Lampit et al. (2014) concluded that unsupervised at-home training regimes were less effective than group-based sessions, and that training more than three times a week was also ineffective. Another important variable was the number of video games used during the training sessions. Although not significant, Toril et al. (2014) found a trend in the analysis indicating that it is better to use a small set of video games than a large set.

It is important to stress that, on the basis of previous results (Ballesteros et al., 2014, 2015b; Lampit et al., 2014), we designed the present study as a group-based training program with the presence of the experimenter throughout. The presence/absence of the experimenter might affect the participants’ interest in training (Borella et al., 2010). It is important to note that in our previous training study (Ballesteros et al., 2014) the experimenter was always present during each training session, but each session involved only 2–3 participants and not the whole group. The lack of improvement after training in visuospatial WM in our previous study (Ballesteros et al., 2014) and the positive results obtained in the present study might be due to the larger number of video games used in the earlier study (10) compared to just six specially selected to train visuospatial WM in the present study. The training regime of the present study was focused on enhancing WM, and this might explain the positive training effects obtained here.

Another important question regarding cognitive training concerns transfer. The evidence of transfer from video game training to untrained tasks is mixed, with both positive and negative results. Some researchers (Melby-Lervåg and Hulme, 2013) argue that WM training has positive effects on tasks close to the trained tasks (near transfer). However, in a more recent study the same authors did not find evidence that WM training was effective (Melby-Lervåg and Hulme, 2016). Owen et al. (2010) examined whether some training tasks would improve cognitive performance, and concluded that there was no transfer to untrained tasks. In our study, we found positive near transfer effects, as well as smaller transfer effects on other untrained episodic memory tasks. Moreover, it is important to stress that there is not an overlap between the training and the transfer tasks.

## Conclusions, Limitations of the Present Study and Future Directions

To summarize, the results of the present study suggest that training older adults with non-action video games can be an effective way of improving visuospatial WM performance in tasks designed to assess this type of memory and episodic memory tests. Importantly, the effects were maintained over a 3-month no-contact follow-up period in the Jigsaw puzzle task, Digit forward (short-term memory), and Faces I and Faces II (episodic memory). Transfer effects were not maintained on Corsi blocks. These findings suggest that older brains retain plasticity, but that some periodic boosting sessions are needed to maintain the benefits.

The present study has several limitations. First, our sample was smaller than in other studies (e.g., Mozolic et al., 2011; Anguera et al., 2013). However, it is important to stress that we did not have any drop-outs, which is unusual in longitudinal training studies, which lose between 30 and 40% of participants at follow-up. This suggests that training programs carried out in places that older adults attend regularly, and training sessions attended by the whole group with the presence of the experimenter are more effective than training individually at home or in small groups. Secondly, we did not examine the effects of training older adults with video games on everyday life tasks. This is an important issue for future studies. Thirdly, the control group in the present study was passive. However, most studies have also used a passive control group (e.g., Goldstein et al., 1997; Basak et al., 2008; Maillot et al., 2012; Ballesteros et al., 2014) to compare with the trained group, only a few training studies involving both an active and a passive control group (e.g., Torres, 2008; Stern et al., 2011; Anguera et al., 2013; Boot et al., 2013a). However, it is worth mentioning that in their meta-analysis, Toril et al. (2014) calculated the effect sizes of the published studies that included both an active and a passive control group (5 out of the 20 studies in the meta-analysis). The mean effect size (Cohen’s *d*) was 0.36 for the active control group and 0.37 for the passive group. The difference was not statistically significant. However, future studies would include active and passive control groups.

It would be interesting in future studies to include a questionnaire to assess expectation (anticipated cognitive gains from game play) and the effects of the social contact with the experimenter and the other older adults. Furthermore, video game designers need to work with researchers in aging to create attractive and useful games specifically designed for older adults. Video games have to be interesting to motivate older adults to play them.

Commercial brain-training programs are currently generating millions of dollars, with very large revenues for the brain-training industry. Video game training is a very active area of research, but there are still important intervention-based factors that require further research. Large-scale longitudinal studies with long follow-up assessments of trained and control groups are necessary before researchers can answer many important questions related to the effectiveness of video-games to improve cognition (Boot et al., 2013b; Boot and Kramer, 2014; Green et al., 2014; Anguera and Gazzaley, 2015).

In conclusion, future research should investigate ways of designing video game training regimes that produce and maintain training benefits in older adults. Further research should take into account multi-domain interventions that can be carried out in social settings, involving computerized cognitive training (e.g., video game training) and physical exercise (Ballesteros et al., 2015b). In sum, future studies would benefit from using well-supported neuroscience findings to design multi-domain, longitudinal intervention studies to investigate the possible benefits for older adults, and then validate the benefits of the intervention.

## Author Contributions

PT, JM, SB and JMR: conceived and designed the experiments, contributed reagents/material/analysis tools, wrote the article, and reviewed the manuscript. PT: performed the experiments, analyzed the data. JMR: reviewing the data. PT, SB, JM: reviewing literature.

## Conflict of Interest Statement

The authors declare that the research was conducted in the absence of any commercial or financial relationships that could be construed as a potential conflict of interest.

## Footnotes

^1^http://www.lumosity.com

^2^The authors confirm that they did not have any contact with the *Lumosity* cognitive training platform at any time during the study, which was conducted independently.
